# Transcriptomic profiling of thymic dysregulation and viral tropism after neonatal roseolovirus infection

**DOI:** 10.3389/fimmu.2024.1375508

**Published:** 2024-06-04

**Authors:** Andrei Belean, Eden Xue, Benjamin Cisneros, Elisha D. O. Roberson, Michael A. Paley, Tarin M. Bigley

**Affiliations:** ^1^ Division of Rheumatology, Department of Medicine, Washington University School of Medicine, St. Louis, MO, United States; ^2^ Division of Rheumatology, Department of Pediatrics, Washington University School of Medicine, St. Louis, MO, United States; ^3^ Department of Genetics, Washington University School of Medicine, St. Louis, MO, United States; ^4^ Department of Pathology and Immunology, Washington University School of Medicine, St. Louis, MO, United States; ^5^ Department of Molecular Microbiology, Washington University School of Medicine, St. Louis, MO, United States

**Keywords:** roseolovirus, thymus, central tolerance, transcriptomics, thymocytes, medullary thymic epithelial cells (mTECs), tropism

## Abstract

**Introduction:**

Herpesviruses, including the roseoloviruses, have been linked to autoimmune disease. The ubiquitous and chronic nature of these infections have made it difficult to establish a causal relationship between acute infection and subsequent development of autoimmunity. We have shown that murine roseolovirus (MRV), which is highly related to human roseoloviruses, induces thymic atrophy and disruption of central tolerance after neonatal infection. Moreover, neonatal MRV infection results in development of autoimmunity in adult mice, long after resolution of acute infection. This suggests that MRV induces durable immune dysregulation.

**Methods:**

In the current studies, we utilized single-cell RNA sequencing (scRNAseq) to study the tropism of MRV in the thymus and determine cellular processes in the thymus that were disrupted by neonatal MRV infection. We then utilized tropism data to establish a cell culture system.

**Results:**

Herein, we describe how MRV alters the thymic transcriptome during acute neonatal infection. We found that MRV infection resulted in major shifts in inflammatory, differentiation and cell cycle pathways in the infected thymus. We also observed shifts in the relative number of specific cell populations. Moreover, utilizing expression of late viral transcripts as a proxy of viral replication, we identified the cellular tropism of MRV in the thymus. This approach demonstrated that double negative, double positive, and CD4 single positive thymocytes, as well as medullary thymic epithelial cells were infected by MRV *in vivo*. Finally, by applying pseudotime analysis to viral transcripts, which we refer to as “pseudokinetics,” we identified viral gene transcription patterns associated with specific cell types and infection status. We utilized this information to establish the first cell culture systems susceptible to MRV infection *in vitro*.

**Conclusion:**

Our research provides the first complete picture of roseolovirus tropism in the thymus after neonatal infection. Additionally, we identified major transcriptomic alterations in cell populations in the thymus during acute neonatal MRV infection. These studies offer important insight into the early events that occur after neonatal MRV infection that disrupt central tolerance and promote autoimmune disease.

## Introduction

The herpesviruses have been hypothesized to promote development of autoimmune diseases, but only recently have investigators found causative evidence of herpesviruses inducing autoimmunity (1–3). For example, studies found that Epstein Barr virus has a causative role in development of multiple sclerosis, likely through molecular mimicry (3–7). Similarly, the human roseoloviruses, HHV-6 and -7, have been associated with autoimmune disease but studies have not identified a clear causal role due to the ubiquitous and chronic nature of roseolovirus infections that typically occur early in life, years before development of autoimmunity (8–14). Murine roseolovirus (MRV) is a natural murine pathogen closely related to the human roseoloviruses that has provided an opportunity to perform mechanistic, *in vivo* studies to understand roseolovirus pathogenesis (2, 15–17). Our studies demonstrated that neonatal MRV infection causes autoimmune disease manifested as autoimmune gastritis and broad autoantibody production (2). Interestingly, this appears to occur through disruption of processes involved in thymocyte survival and central tolerance. We observed that MRV induces depletion of CD4 single positive (CD4SP) and CD4+CD8+ double positive (DP) thymocytes, reduction in medullary thymic epithelial cell (mTEC) numbers, and disruption of tissue restricted antigen (TRA) and *Aire* expression (2). Our findings presented a unique paradigm of virus-induced autoimmunity in which early life viral infection of the thymus can produce durable immune dysregulation that leads to autoimmunity.

Indeed, multiple pathogens can infect the thymus (18, 19). While infection of the thymus by several different viruses has been shown to result in thymic atrophy, thymocyte depletion, altered expression of genes that contribute to thymic function, or direct infection of thymic stromal cells, only MRV has been shown to induce autoimmunity (2, 18–29). Human roseoloviruses have been shown to infect human thymus xenografts in SCID mice and induce thymic atrophy, resulting in a reduction in DP and CD4SP cells. These studies, however, did not evaluate for the subsequent development of autoimmunity after resolution of acute infection (30). The impact of human roseolovirus infection on the thymus *in vivo* in humans is largely understudied, although there exists some evidence that acute human roseolovirus infection occurs in patients receiving a thymus transplant (31, 32). A better understanding of how thymotropic viruses, including the roseoloviruses, impact thymic function and tolerance requires further evaluation of host-virus interactions at a molecular level as well as identification of viral tropism in the thymus.

Central tolerance is a complex process in which millions of dividing thymocyte progenitors must form a functional T cell receptor (TCR) and receive the appropriate signals to survive (33, 34). This begins at the stage of the early thymic progenitors (ETP) that arrive from the blood. Notch signaling and the activity of key T-cell transcription factors such as *Tcf7*, promote proliferation and differentiation of ETPs into double negative (DN) thymocytes that lack CD4 and CD8 expression (35–42). DN cells receive further stimulation through Notch and other signaling events that promote progression through four DN stages that include highly proliferative DN1 and DN2 stages, followed by a DN3 stage in which the TCRβ locus rearranges to form a pre-TCR (43–48). Signaling through the pre-TCR, Notch signaling, and activity of a host of transcription factors allows progression to the DN4 stage followed by maturation to the molecularly distinct immature single positive (ISP) stage in which CD8 is expressed (34, 38, 45, 49–52).

Following the ISP stage, cells upregulate expression of CD4 and CD8, and are called double positive cells (DP). Single-cell transcriptomics has identified that DP cell development is characterized by three distinct stages (43, 53). This includes the DPbla (blast) stage that occurs after the ISP stage and is characterized by rapid proliferation, the DPre (rearranging) stage in which the TCRα locus is rearranged and the mature TCR can receive signal for positive selection, and the DPsel (selection) stage when cells undergo positive selection (53). Positive selection is mediated by antigen presenting cells called cortical thymic epithelial cells (cTECs) that express self-MHC molecules for DP thymocytes to sample TCR binding for sufficient affinity to promote survival (54, 55). Upon completion of positive selection, DP thymocytes downregulate either CD4 or CD8 to become CD4SP or CD8 single positive (CD8SP) and migrate to the thymic medulla. Negative selection then occurs through a process of affinity-based selection in which MHC:self-antigen on mTECs and thymic dendritic cells is presented to CD4SP and CD8SP cells, although evidence exists suggesting that some negative selection can also occur at the DP stage in the cortex or corticomedullary junction (55–57). Expression of self-antigen (aka TRAs) is driven by key transcription regulators such as Aire and Fezf2 (58). Cells that survive positive and negative selection migrate out of the thymus. This process requires intricate and coordinated signaling and interactions that, if disrupted, can alter T cell development and lead to immune deficiency and/or autoimmunity (54, 58–61).

Like all herpesvirus infections, beta-herpesvirus infection features complex virus-host interactions in which cellular machinery is subverted to support viral replication and modulate the immune response. Many of the cellular processes that are important for thymocyte development are altered during beta-herpesvirus infections, although there are few studies assessing the impact of herpesvirus infections in the thymus at the molecular level (2, 15, 16, 62, 63). One important cellular process that is influenced by herpesviruses is the cell cycle. The beta-herpesviruses, which includes cytomegaloviruses and roseoloviruses, induce an arrest of the cell cycle at the G1/S interphase while also inducing activation of cell cycle machinery, DNA damage response, and apoptosis pathways necessary for viral DNA replication (64–68). The beta-herpesviruses modulate multiple aspects of the immune response including epigenetic silencing, interferon and cytokine signaling, proteasome and inflammasome response, and antigen presentation (66, 69–71). Host transcription and translation machinery are hijacked to promote viral gene and protein expression, while cellular metabolism is altered to support viral replication (72, 73). For example, lipid metabolism is reprogrammed during beta-herpesvirus infection and plays a role in cellular and viral membrane formation during infection (74). Herpesvirus infection can also impact cellular differentiation. For example, the beta-herpesviruses have been shown to alter neuronal and hematopoietic cell differentiation through direct infection and by altering the microenvironment and signaling such as the Notch signaling pathway (75–79). The complex interaction between herpesviruses and cellular processes illustrate potential molecular mechanisms by which direct infection and bystander effects could disrupt thymic functions such as thymocyte development and induction of tolerance.

Transcriptomics have been utilized to better understand herpesvirus-host interactions at a molecular level. Although the cellular tropism of the beta-herpesvirus is wide, single-cell transcriptomics studies have predominantly focused on infection of specific cell types *in vitro* or *ex vivo* (80–83). This has allowed for evaluation of infection kinetics and virus-host interactions in specific cell types. These studies have provided important insight into lytic and latent infection and cellular pathways altered by infection. Herpesvirus gene expression follows a kinetic pattern that is temporal and coordinated through three phases: immediate early (IE), then early (E), followed by late (L), although a set of genes are referred to as E/L (also called leaky late) that have mixed E and L kinetics (84). While initiation of infection is characterized by IE gene expression, productive viral genome replication is dependent on E genes, and robust expression of L genes that contribute to virion assembly is dependent on viral genome replication (84, 85). Bulk and single-cell transcriptomics have established patterns of expression in different cells and at different times post-infection, providing information about the expression pattern of uncharacterized viral genes (80, 82, 86, 87). Single-cell transcriptomics during acute, *in vivo* herpesvirus infection of an entire organ, such as the thymus, has not been performed to date but offers the opportunity to identify tropism of the virus and establish how infection disrupts normal cellular processes in infected and surrounding cells.

In this study, we examined the single-cell transcriptome of the entire thymus during acute MRV infection and compared these findings to an uninfected thymus on day of life (DOL) 6. We simultaneously assessed both host gene expression and viral gene expression patterns. We explored the tropism of MRV in the thymus, which includes thymocytes at the DN3, DN4, ISP, DP, and CD4SP. We also assessed the differential cellular gene expression patterns that defined the uninfected and infected thymus, including those that are important for thymocyte development and herpesvirus replication. Moreover, our studies evaluated MRV infection of mTECs and expression of genes that may impact interactions between antigen presenting cells (i.e. mTECs) and thymocytes. We used computational approaches to explore the kinetics of the viral transcriptome by cell type and infection status. Finally, we applied our *in vivo* tropism findings to establish cell culture systems that are susceptible to MRV replication. Our findings demonstrate the utility of single-cell transcriptomic approaches to study complex virus-host interactions as well as viral tropism within an entire organ *in vivo*.

## Materials and methods

### Mice and infection

BALB/c mice were purchased from Charles River Laboratories. Mice were bred in-house in specific pathogen free facilities. MRV infected mice were housed separately from uninfected mice to avoid horizontal transmission. We conducted our studies in accordance with the institutional ethical guidelines through institutional animal care and use committee (IACUC) protocol that was approved by the Animal Studies Committee of Washington University. Mice were infected with MRV as previously described using stock from *in vivo* passaging (15, 17, 63). Briefly, in day of life (DOL) 0, mice were injected with 1e8 viral genomes via intraperitoneal (i.p.) injection.

### Flow cytometry

Analysis of non-stromal thymocytes at 3, 5 and 7 days post infection (dpi) was performed by dissecting the thymus and mincing into small pieces with scissors. Pipetting with a large bore pipette was used to liberate thymocytes. Cells were prepared for flow cytometry by staining in a fixable viability dye (eBioscience) and blocking the Fc receptor with 2.4G hybridoma supernatant (made in house). Surface staining was performed before fixation and permeabilization with Foxp3/Transcription Factor Staining Buffer Set (eBioscience). Florescent-labeled antibodies used in this study included: anti-CD4 (RM4–5) and anti-CD8α (53–6.7) from Fisher Scientific; anti-CD19 (6D5) and anti-NKp46 (29A1.4) from Biolegend; anti-CD45.2 (88) from eBioscience. Flow cytometry was performed using a FACSCanto (BD Biosciences) and analyzed using FlowJo v10 (TreeStar, Ashland, OR).

### Preparation of cells for scRNAseq

The thymus was dissected with care to remove non-thymic tissue using scissors. The thymus tissue was placed into 1ml of 0.25% trypsin EDTA (GIBCO) at 37°C. The tissue was incubated for 15 minutes. Dissociation and digestion were aided by vigorous pipetting using a large bore pipette every 5 minutes. Cells were filtered through a 100μm filter into 9ml of RPMI 10% FBS to neutralize trypsin activity. Cells were then centrifuged at 1500rpm for 3.5 minutes followed by incubation in RBC lysis buffer (made in house) for 3 min at room temperature. Cells were then centrifuged again and washed twice in 9ml of PBS 0.5% bovine serum albumin (BSA). Cells were counted via hemocytometer then resuspended at 1200 cells/μL in PBS 0.04% BSA. The cells were kept on ice until ready for scRNAseq analysis.

### Library preparation and 3’ scRNAseq

Single-cell 3’ gene expression cDNA libraries were generated per manufacture protocols using the 10X Genomic Chromium Single-Cell 3’ Library and Gel Bead Kit v1 and the 10x Chromium Controller (10x Genomics, Pleasanton, CA) platform for microdroplet-based, single-cell barcoding. This was performed by the Genome Technology Access Center at the McDonnell Genome Institute (GTAC@MGI, Washington University in St. Louis). Libraries were sequenced at the GTAC@MGI on the NovaSeq Sequencing System (Illumina, San Diego, CA).

### scRNAseq analysis

Raw reads were processed with Cell Ranger (v3.1.0). The raw reads were initially mapped to the mouse genome. Any remaining unmapped raw reads were then mapped to the MRV genome. These formed two gene count matrices used for analysis. Docker containers were used for reproducibility, with two different containers being used for different parts of the analysis. Primarily, abelean/seurat_desctools:4.1.0 was used, and abelean/seurat_monocle:4.1.1 was used for all scripts that included Monocle3. Data were analyzed in R (desc: 4.1.0, m3: 4.2.1) using the Seurat package (desc: 4.9.9.9044, m3: 4.3.0) and Monocle3 (m3: 1.2.9), the latter of which was used for pseudotime analysis. Scripts were run on the Washington University in Saint Louis RIS Scientific Compute Platforms.

Dimensionality reduction was performed using SCT integration in Seurat, using Mock as a reference, with the *FindIntegrationAnchors*, *IntegrateData, and RunUMAP* functions on default settings. Unbiased clustering was performed with *FindNeigbors* and *FindClusters*. A resolution of 1.0 was used in *FindClusters* based on the separation of cell types using canonical gene expression (53, 89–97). The data was split into indicated cell types with the *subset* function. The subsetting was based on the annotations used for the clustering, which were produced by labeling the clusters from *FindClusters* with the *AddMetaData* function. The annotations were made based on Feature Plots of canonical gene expression. Specific cell types were subsetted and had dimensionality reduction repeated for downstream analysis.

With the dimensionality reduction of certain data subsets (TECs, DCs, etc.), SCT integration did not distinguish cell types with a small sample size. Instead, the Harmony (desc: 0.1.1) package was used, using the *RunHarmony* function, which allowed for the resolution of these distinct cell types. For identifying differentially expressed genes (DEGs), *FindAllMarkers* was used with the *Idents* function used to select the clustering, followed by heatmaps with *DoHeatmap*. We selected markers based on the following criteria: only positive, minimum percent expressed ≥ 0.25, logFC ≥ 0.25, and adjusted p-value < 0.05. The package EnrichR (desc: 3.0) was used to calculate pathway enrichment based on the DEGs, using the *enrichr* function. Bar plots were generated using the ggplot2 (desc: 3.4.2, 3.4.0) package. The hierarchical heat map was generated with the pheatmap (desc: 1.0.12) package.

The package nichenet (desc: 1.1.1) was used to calculate interactions between specific cell types. Nichenet utilizes selected receiver and sender(s) from metadata, for which we used cell type annotations. Next, the gene expression between receiver and sender(s) is run through a database of potential ligand-receptor interactions. We examined was the DP or SP cells as the receivers, and cTECs or mTECs/DCs as senders, respectively.

*ORF Analysis:* Criteria for “infected” cells and “replicating” cells were as follows: infected cells were defined as having expression of at least one of each of the following ORFs: *ORF55, ORF83, and ORF103*, and replicating cells were defined as having expression of least three copies of four out of five of the following ORFs: *ORF45, ORF53, ORF69, ORF73*, or *ORF86*. For dimensionality reduction based on ORFs, barcodes with 0 reads were excluded. For “pseudokinetics” analysis with Monocle3, the package SeuratWrappers (0.3.0) was used to convert Seurat objects into a cell dataset format using *as.cell_data_set*. Next, the object was run through Monocle3, with *learn_graph* and *order_cells* functions used to generate the pseudotime. With the nodes produced by *learn_graph*, the node corresponding to the earliest biological stage was selected, i.e., the infected stage in the “infected vs. replicating” analysis. For the pseudo kinetics, ORFs with 0 gene counts were converted to 0.1 values for visualization on a log scale.

### Cell culture, infection, and nucleic acid analysis

Cells were all cultured under sterile conditions and incubated at 37°C. MOHITO cells are a non-adherent, CD4+CD8+ T cell lymphoblastic leukemia cell line derived from a sublethally irradiated female BALB/c mouse (98). MOHITO cells were propagated in Prigrow II media with 20% FBS, 10ng/ml of recombinant mouse IL-7, and 1% Penicillin/Streptomycin (Abm). Media was changed every 2–3 days with retention of 20% conditioned media and cells were maintained at 4–8e5 cells/mL. Thymocytes (non-adherent) were dissected from day of life 7 mice by dissecting the thymus and mincing with scissors. After dissociation of thymocytes by pipetting, stromal debris was allowed to settle, and supernatant was passed through a 70μm filter. Cells were propagated in RPMI 10% FBS, 2 mM L-glutamine, 1 mM sodium pyruvate, 100 mM non-essential amino acids, 5 mM HEPES free acid, 10 ml of 5.5 × 10−2 2-mercaptoethanol, and 100 U/ml Penicillin/Streptomycin (Gibco). Media was changed every 2–3 days with retention of 33% conditioned media and cells were maintained at 0.5–1e6 cells/mL. mTE4–14 cells are adherent, spontaneously immortalized cells derived from primary mTEC culture isolated from the thymi of neonatal C3H/J mice (99). mTE4–14 cells were grown in DMEM 10% FBS, 1% Penicillin/Streptomycin (Gibco). Cells were grown on tissue culture treated plates, split using trypsin when confluent and replated at ~70% confluency. Media was changed every 2–3 days. Primary mTECs were harvested from day of life 7 BALB/c mice as described previously by dissecting a thymus and mincing it with scissors, followed by several rounds of vortexing and removal of supernatant to reduce the number of thymocytes (100, 101). Stromal pieces were then plated and mTECs were allowed to migrate onto the plate for 3–4 days. Media was changed with care not to disrupt adherent cells and to leave thymus pieces while removing remaining thymocytes. Media was changed every 2–3 days and after 10 days, remaining thymic debris was removed. Cells were split using trypsin. Primary mTECs were propagated in cFAD media: DMEM/Ham’s F12 1:1 (Gibco), 5% FBS, Insulin 3μg/mL (Sigma), Cholera toxin 10ng/mL (Sigma), epidermal growth factor 20ng/mL (Sigma), hydrocortisone 0.5μg/mL (Sigma), adenine 24μg/mL (Sigma), 1% Penicillin/Streptomycin (Gibco). Flow cytometry was used to verify expression of expected cell surface markers for each cell type (MOHITO culture: CD4+CD8+; Thymocyte culture: CD4 and CD8 to identify DN, DP, and SP populations; mTE4–14 and primary mTEC culture: EPCAM+UEA1+Ly51-).

Cells were counted via a hemocytometer before infection and 2.5e5 cells were added per well. Cells were then infected with 1 genome/cell as determined by qPCR analysis of *in vivo* stocks prepared as described above (2). 24 hours post infection, cells were washed with PBS and media was replaced. Media was changed every 3–4 days. Cells were collected by centrifugations for non-adherent cells and by trypsin digestion for adherent cells. Cell counts were performed using a hemocytometer and recorded before storage of cells to ensure known cell counts per sample to calculate number of genomes per cell and for graphical representation as MRV genomes per 2.5e5 cells.

DNA was prepared from cell culture using the QIAAmp Kit (Qiagen). qPCR of DNA was performed in technical duplicate using Taqman Universal Master Mix II (Applied Biosystems) on a StepOnePlus or a QuantStudio 3 real-time PCR machine (Applied Biosystems). MRV *ORF69* was quantified using a plasmid of known base pair number containing the *ORF69* gene, which was used as a standard curved and to calculate number of genomes in the sample. Primers used included ORF69 (5′-CAA​GTC​TGA​TTG​AGG​ATT​CAC​TTT​ATG-3′, 5′-56-FAM/TCCAAATCC/ZEN/ACAATTCCCGTCTCTGT/3IABkFQ-3′, and 5′-CGT​CGA​TAG​TTG​GCA​AGA​AGA-3′).

### Statistical analysis

The data were analyzed for statistical significance using GraphPad Prism 10. We used multiple Student’s *t*-test for multiple comparison of 2 groups. Statistical significance was denoted as * for *p* < 0.05. Comparisons without significance are not marked except. Error bars represent standard deviation for all graphs.

### Data and code availability

All sequencing data is available on the NCBI Gene Expression Omnibus under accession GSE255738. Code will be available at https://github.com/abelean/MRV-R-analysis.

## Results

### Transcriptomics identified MRV tropism in the thymus during neonatal infection

**Figure 1:**
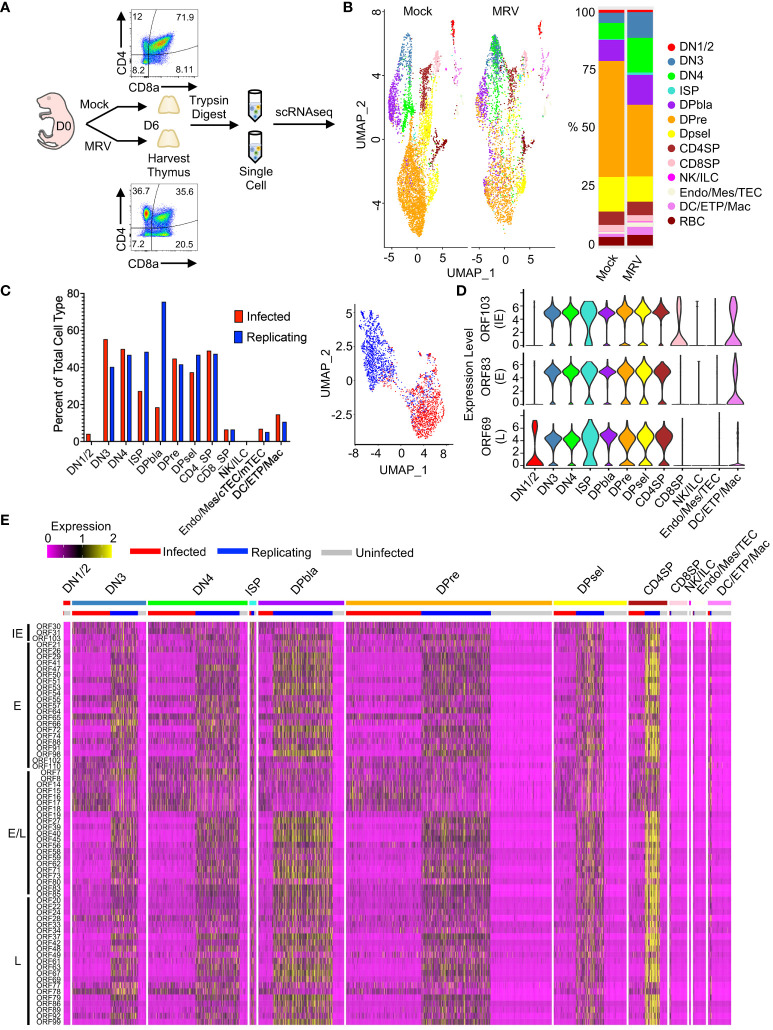
scRNAseq identified MRV tropism in the thymus after neonatal infection. **(A)** Overview of experimental setup: Mock or MRV infection was performed on day of life (DOL) 0. At 6 days post infection (dpi) the thymus was collected. Mock and MRV infected thymuses were processed in parallel. Flow cytometry was performed to demonstrate depletion of DP population on DOL 6. The thymus as digested with trypsin to obtain single-cell suspensions that were submitted for scRNAseq. **(B)** UMAP of thymocytes split by infection (Mock vs MRV, left panel), and proportions of identified cell types (right panel). **(C)** Proportion of “Infected” and “Replicating” cells within MRV-infected samples (see Methods for defining ORFs) (left panel). UMAP of infected and replicating cells defined by MRV ORF expression (right panel). **(D)** Violin plots of key canonical ORF expression by cell type. **(E)** Heat map of ORF expression, ordered by stages of thymic development and infection status (infected/replicating/uninfected). ORFs were ordered by putative kinetics based on homology to HHV-6 and HCMV: immediate early (IE), early (E), early/late (E/L), and late (L).

We utilized single-cell RNA sequencing (scRNAseq) to identify MRV tropism in the thymus. At 7dpi, there was prominent depletion of CD4+ single positive (CD4SP) and CD4+CD8+ double positive (DP) thymocytes, while at 3 and 5 dpi there was minimal or partial reduction in CD4SP and DP populations (**Supplementary Figure 1A**) (15, 17). At 6dpi, CD4SP and DP depletion is detected compared to mock infected mice, but there remained an adequate number of both populations for analysis (**Figure 1A**). We therefore performed scRNAseq of the entire thymus of BALB/c mice after mock or MRV intraperitoneal infection at DOL 0. At 6dpi the thymus was dissected from three individual mice, digested to single cell suspension, combined into a single sample, then submitted for scRNAseq (**Figure 1A**).

Unbiased clustering of mock vs MRV infected thymus demonstrated populations of cell types expected to be found in the thymus, including thymocytes at different stages of development, i.e. the double negative (DN), DP, and single positive (SP) stages, as well as natural killer cells or innate lymphocyte (NK/ILC), endothelial/mesenchymal cells, thymic epithelial cells (TEC), dendritic cells (DC), early thymic progenitors (ETP), and macrophages/monocytes (**Figure 1B**). We identified alterations in the percent and number of various populations after MRV infection, notably a relative reduction in the DP populations after MRV infection (**Figure 1B**). To differentiate cells that were uninfected, MRV infected, or infected and supporting MRV replication (referred to as “Uninfected,” “Infected,” and “Replicating” henceforth) we utilized known patterns of herpesvirus gene expression to establish a criterion to define tropism and the stage of viral replication cycle. Putative MRV ORF kinetics were assigned based on homology to HHV-6 and HCMV. Cells expressing at least one transcript of *ORF55* (E), *ORF83* (E), and ORF103 (IE), but not demonstrating robust expression of early/late or late genes were labeled as “Infected.” Cells with robust expression of all stages of ORF expression, which would suggest MRV replication, were labeled as “Replicating” [i.e., expressing at least three copies (unique molecular identifiers) of 4 out of 5 of the following ORFs: *ORF45* (E/L), *ORF53* (E), *ORF69* (L), *ORF73* (E/L), or *ORF86* (L)] (82, 84). We then identified the percentage of cells that were MRV infected or replicating, and found that DN3, DN4, DP, immature single positive (ISP), and CD4SP all supported infection and replication in >25% of cells, while lower levels were observed in other cell populations (**Figure 1C**). UMAP dimensional reduction using viral ORF expression of cells designated as either infected vs replicating demonstrated two clear transcriptional clusters, supporting our approach to differentiating these stages of infection (**Figure 1C**).

Evaluation of representative viral ORF expression from IE [*ORF103* (HCMV *UL122*/*123* and HHV-6 *U86* homologue)], E [ORF83 (HCMV *UL97* and HHV-6 *U69* homologue], and L [*ORF69* (HCMV *UL82*/*83* and HHV-6 *U54* homologue)] stages in each cell type showed robust expression in cell types classified as replicating (**Figure 1D**). We next identified patterns of expression in each cell type, differentiated by uninfected, infected, or replicating. We found that cells designated as supporting MRV replication demonstrated robust expression of most MRV ORFs, with CD4SP showing the highest relative ORF expression (**Figure 1E**). In contrast, the relative expression of IE *ORF30* and *ORF31* (HCMV *UL47* and *UL38* respective homologues, HHV-6 *U30* and *U19* homologues), *ORF14, 15, 16, 17, 18* (US-22-like), and *ORF78* (HCMV *UL93* and HHV-6 *U64* homologue) were increased in cells classified as MRV infected compared to replicating (**Figure 1E** and **Supplementary Figure 1B**). These data establish the pattern of MRV thymic tropism after neonatal infection with DN3, DN4, DP, ISP, and CD4SP infected at a high percentage, while lower percentage of infection occurred in other cell types. Moreover, we identified distinct patterns of MRV ORF expression at different stages of infection.

### Neonatal MRV infection disrupts DN thymocyte expression of inflammatory, apoptosis, and pluripotency genes

**Figure 2:**
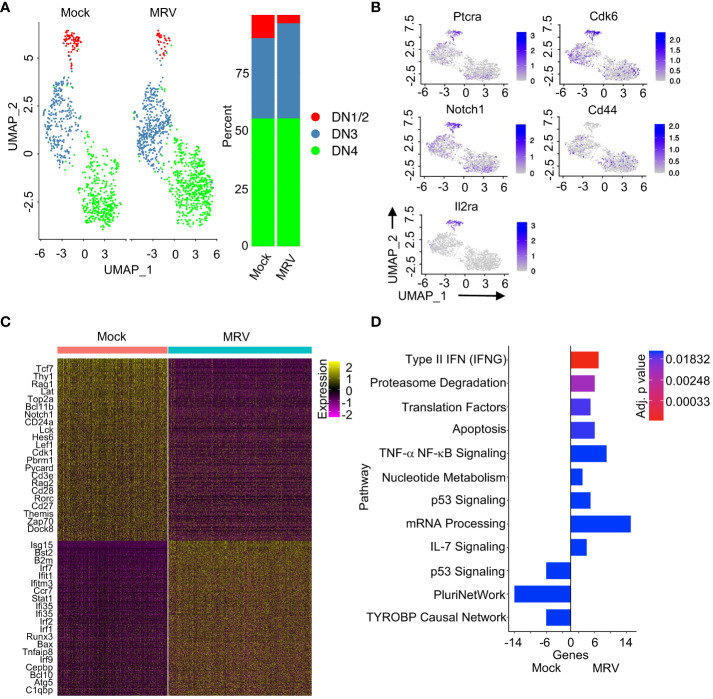
Neonatal MRV infection disrupts DN thymocyte transcription. **(A)** UMAP of the DN populations split by infection (left panel) and proportions of identified cell types (right panel) are shown. **(B)** Expression of indicated DN marker genes are shown on UMAP. **(C)** Heat map of DEGs for DN thymocytes between Mock vs. MRV. Relevant genes are displayed. **(D)** Enrichment of indicated pathways in DN thymocytes for Mock and MRV. + genes are upregulated in MRV and – genes are upregulated in Mock.

Specific cell surface and transcriptional markers have been used to differentiate the DN stages (**Figure 2B**) (43). We found that at 6dpi, neonatal MRV infection resulted in a modest relative reduction in DN1/DN2 cell number and percentage, and an increase in the relative number of DN3 cells (**Figure 2A**). Evaluation of differentially expressed genes (DEGs) demonstrated considerable transcriptomic alterations after MRV infection. Genes that play important roles in DN cell development and signaling (i.e. *Tcf7, Lat, Bcl11b, Notch1, Lck, Hes6, Rorc, Themis, Zap70 and Dock8*), cell cycle (*Cdk1*), and TCR rearrangement (*Rag1, Rag2*) were downregulated by MRV infection. Genes involved in interferon signaling (i.e. *Isg15, Irf7, Ifit1, Ifitm3, Stat1, Irf1 and Irf2*) as well as tumor necrosis factor (TFN) signaling and apoptosis/autophagy (*Bax, Tnfaip8, Bcl10, Atg5*) were upregulated during MRV infection (**Figure 2C**).

Pathway analysis confirmed that neonatal MRV infection induced upregulation of genes involved in type II IFN, proteasome degradation, translation, apoptosis, TNF signaling, mRNA processing, and IL-7 signaling pathways (**Figure 2D**). Of note, there were genes involved in p53 signaling pathways that were either up- or downregulated by infection (**Figure 2D**). Many of these pathways have been demonstrated to be manipulated by herpesvirus infections. Interestingly, neonatal MRV infection resulted in downregulation of genes involved in pluripotency (PluriNetWork) (102) and the TYROBP pathway that is associated inflammation (TYROBP causal network) (**Figure 2D**) (103). Pathway analysis of each DN population demonstrated relatively less transcriptomic alteration in DN1/DN2 cells compared to alterations induced by MRV in DN3 and DN4 which was characterized by type II IFN, proteasome degradation, cell cycle, and metabolic pathway dysregulation (**Supplementary Figures 2A–C**). The DN3 population demonstrated downregulation of Delta-Notch and pluripotent signaling pathway genes, both of which are involved in DN cell differentiation (**Supplementary Figures 2A–C**) (35, 45). These data provide insight into how MRV disrupts thymocyte development through alteration of expression of genes that contribute to DN cell differentiation, TCR formation, cell cycling, and inflammation.

### Neonatal MRV infection induces IFN and cytokine signaling pathways, and disrupts cell cycle and cholesterol metabolism in DP and SP cell populations

**Figure 3:**
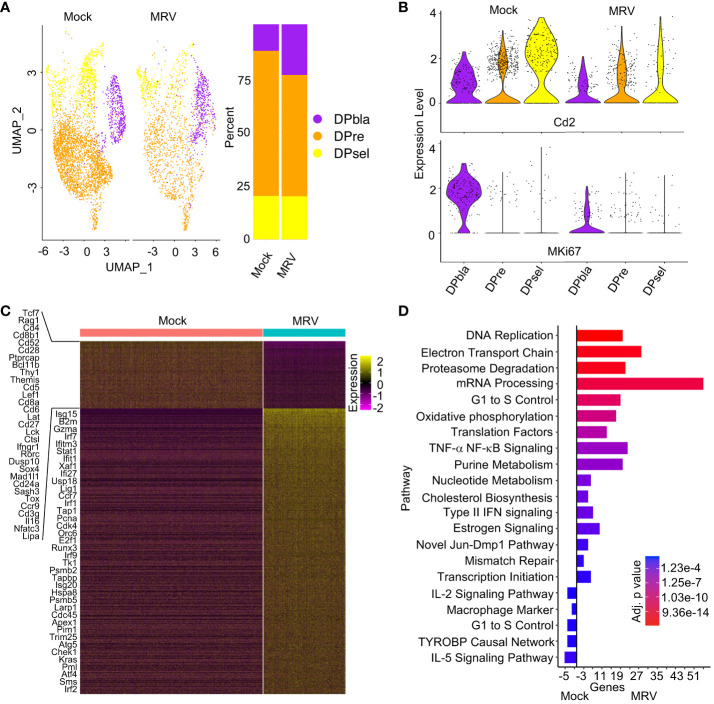
Neonatal MRV infection results in broad transcriptomic alteration in DP cells. **(A)** UMAP of the DP populations split by infection (left panel) and proportions of identified cell types (right panel). **(B)** Violin plots of *Cd2* and *Mki67* for each DP population split by infection. **(C)** Heat map of DP populations finding DEGs between Mock vs. MRV, representative genes are shown. **(D)** Enrichment of indicated pathway in DP thymocytes for Mock vs MRV.

We next evaluated the impact of MRV on the three DP stages of thymocyte maturations (DPbla, DPre, and DPsel) (53). We observed an increase in the relative number of DPbla and reduction in the relative number of DPre thymocytes after MRV infection (**Figure 3A**). Expression of Ki67 and CD2 has been used at a transcriptional and protein level to differentiate the DP stages (53). As has been previously demonstrated, we found the highest expression of *Mki67* in DPbla cells and the highest expression of *Cd2* in DPsel cells (**Figure 3B**). Expression of both markers was decreased by MRV infection (**Figure 3B**).

As observed in DN cells, MRV infection resulted in downregulation of genes that contribute to thymocyte and TCR development including *Tcf7, Rag1, Cd4, Cd8, Cd28, Bcl11b, Thy1, Themis, Cd5, Lat, Cd27, Tox*, and *Cd3g* (**Figure 3C**). As expected, there was upregulation in genes involved in IFN signaling (**Figure 3C**). Pathway analysis of all DP cells demonstrated MRV-associated upregulation of pathways known to be manipulated by herpesvirus infections including DNA replication, proteasome degradation, mRNA processing, cell cycle, and cholesterol biosynthesis (**Figure 3D**) (66). TYROBP causal network genes were downregulated by MRV infection, as were IL-5 signaling pathway genes (**Figure 3D**). Despite DPbla displaying the highest percent of cells meeting cutoff for supporting MRV replication among the DP populations, there were the fewest observed DEGs and pathway alterations. The DPbla population demonstrated the greatest downregulation of cell cycle control and pluripotency genes after MRV infection (**Supplementary Figure 2D**). DPre and DPsel demonstrated similar DEG and pathway changes, which included altered expression of cell cycle and DNA replication, metabolism, IFN and cytokine (IL-2, IL-5), and pluripotency pathway genes (**Supplementary Figures 2E, F**).

**Figure 4:**
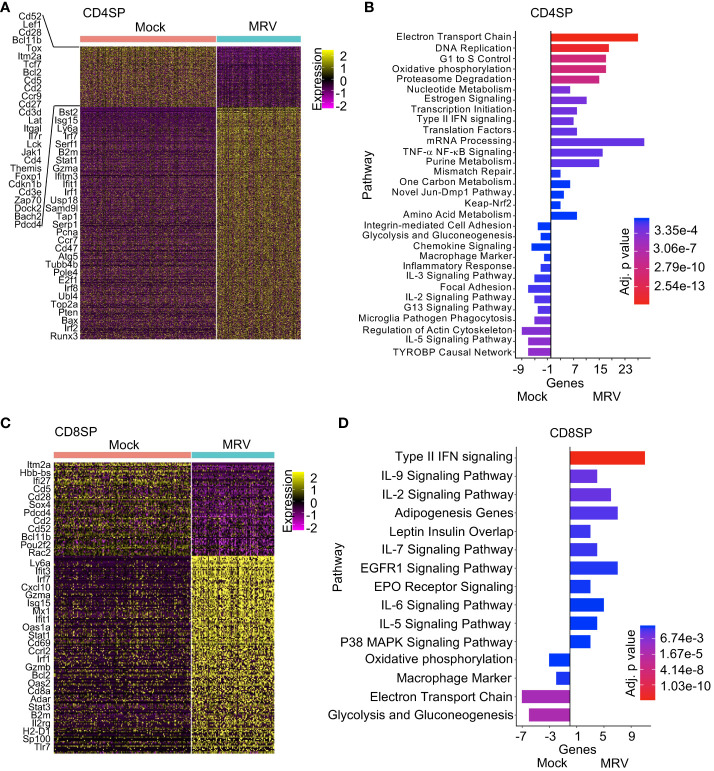
Pathway analysis of CD4SP and CD8SP identifies differential response to neonatal MRV infection. **(A, C)** Heat map analysis of DEGs of Mock vs. MRV with representative genes shown. **(B, D)** Enrichment of indicated pathways in SP thymocytes for Mock and MRV.

A characteristic feature of neonatal MRV infection is thymic and peripheral CD4+ thymocyte and T cell depletion (2, 15–17). Indeed, CD4SP thymocytes demonstrated robust expression of IE, E and L genes and, likewise, broad transcription dysregulation (**Figure 4A**). Pathway analysis revealed upregulation of metabolic pathways (i.e. electron transport, oxidative phosphorylation, proteasome, estrogen signaling, one carbon, animo acid pathways), DNA replication and cell cycle pathways, mRNA and translational pathway, mismatch repair, type II IFN, and TNF-α pathway genes (**Figure 4B**). As in other thymocyte populations, MRV infection was associated with TYROBP signaling, but several other pathways were also downregulated after MRV infection in CD4SP thymocytes, including cell adhesion, glycolysis, chemokine and cytokine signaling (IL-3, IL-2, and IL-5), and cytoskeleton regulation pathway genes (**Figure 4B**). In contrast to CD4SP thymocytes, there were few CD8SP thymocytes demonstrating MRV transcripts to suggest infection or replication (**Figure 1E**). We did observe differential transcriptomic profiles in the CD8SP population when comparing mock vs MRV (**Figure 4C**). These transcriptome differences demonstrated activation of type II IFN and cytokine (IL-9, IL-2, IL-7, IL-6 and IL-5) pathways, while we did not observe differences in genes involved in cell cycle, metabolic, and nucleic acid pathways that were altered in MRV susceptible populations (**Figure 4D**).

We then utilized Immune Response Enrichment Analysis to evaluate the cytokine signature of DN, DP, CD4SP and CD8SP cells, which is based on the data collected by Cui, et al. in which transcriptional responses to individual cytokine stimulation were measured (104). For all cell types, the dominant enrichment included IFN-α1, IL-36a, and IFN-β, although IFN-γ, IL-7, IL-18, IL-15, NP, IL-12, TL1a and IL-2 were also enriched (**Supplementary Figure 3**). For CD8SP, IFN-α1and IFN-β were considerably more represented compared to DN, DP and CD4SP. Finally, we evaluated expression of cytokine transcripts and found that the majority of *Ifng* expression occurred in NK/ILCs, *Il18* expression in DCs, Endo/Mes cells, ETPs, and monocytic cells, and expression of *Il7, Il15*, and *Tnfsf15* in mTECs. Overall, this data demonstrates the pattern of cytokine expression and response in the thymus after MRV infection.

**Figure 5:**
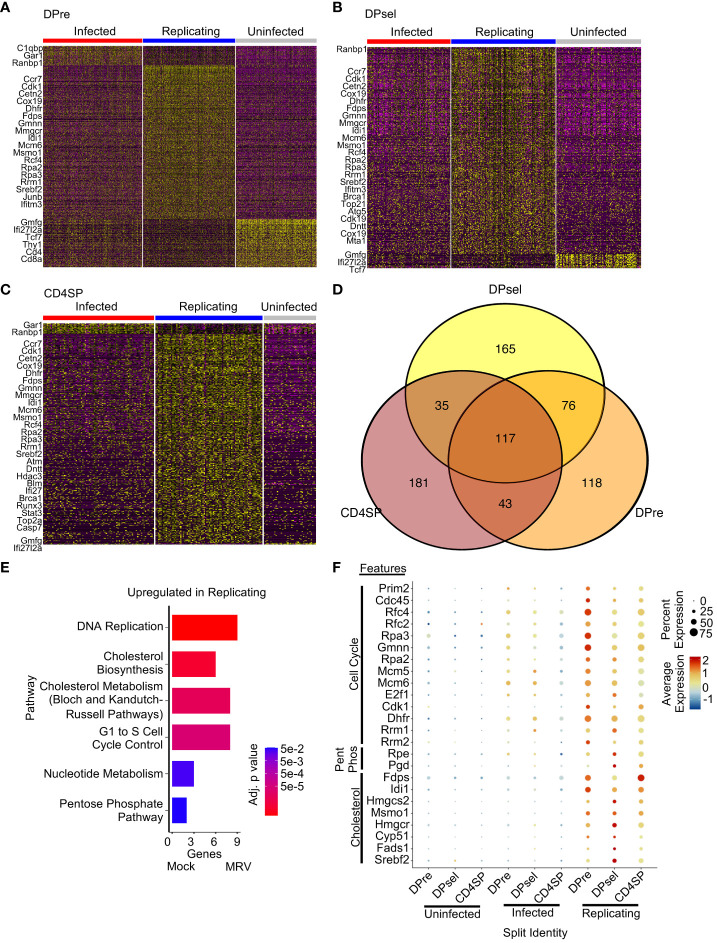
Overlap of transcription disruption after MRV infection by infection status reveals shared features of cells supporting MRV replication. Heatmaps of DEGs between infected, replicating, and uninfected cells for DPre **(A)**, DPsel **(B)**, and CD4SP **(C)**. Representative genes for each group are shown. **(D)** Venn Diagram of DEGs from the replicating clusters were then compared. **(E)** Pathway analysis of replicating related DEGs that are present in at least 2 cell types. **(F)** Dot plot analysis of genes representing pathway changes by cell population and infection status (Pent Phos = Pentose Phosphate Pathway).

The DPre, DPsel and CD4SP populations exhibited comparable numbers of cells that were MRV infected, replicating, or uninfected (**Figure 5**). We therefore sought to identify upregulated genes associated with the viral replication cycle that were shared across distinct cell types. We found 117 genes that were upregulated in cells assigned to the MRV replicating group in all three cell populations (**Figure 5D**). Pathway analysis showed that genes involved in DNA replication, cholesterol metabolism, cell cycle control, and nucleotide metabolism were upregulated (**Figures 5E, F**), demonstrating a cell-intrinsic effect of viral replication on the transcriptional regulation of these pathways. Importantly, pathways that are known to be affected by herpesvirus infections and are crucial to replication were represented in cells supporting MRV replication. Taken together, these data demonstrate a transcriptomic signature of MRV infection and replication characterized by virus-mediated disruption of pathways involved in cell cycle control, nucleotide metabolism and DNA replication, and cholesterol metabolism in DP and CD4SP thymocyte populations.

### DC and mTECs appear susceptible to infection and demonstrate transcriptional aberrations in genes involved in thymocyte development and interaction

**Figure 6:**
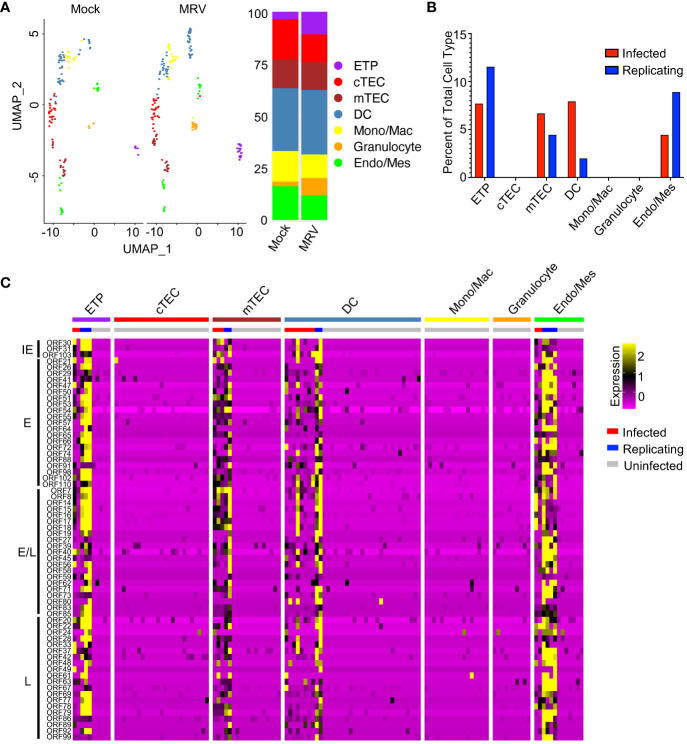
MRV infection of non-thymocyte cells includes mTECs, DCs, and ETPs. **(A)** UMAP and proportion graphs (left and right panel, respectively) of the non-thymocyte cells. **(B)** Percent of MRV cell types demonstrating MRV ORF expression suggestive of MRV infected or replicating. **(C)** Heat map of ORFs as in **Figure 1E**.

Thymocytes constitute most of the cells in the thymus, but other cell types play crucial roles in thymic function. Subsetting and clustering of these minority cell populations in our samples revealed the presence of cTECs, mTECs, DCs, endothelial and/or mesenchymal cells (Endo/Mes), ETPs, granulocytes, and monocytic cells (**Figure 6A**). We observed an increased percentage of ETP, granulocytes, and monocytes/macrophages after MRV infection (**Figure 6A**). Based on MRV gene expression, a minority of endo/mes, ETPs, and mTECs appear to support infection and replication, a minority of DCs appear to be infected without replication, and cTEC, granulocytes, and monocytes/macrophages do not support infection or replication (**Figures 6B, C**).

**Figure 7:**
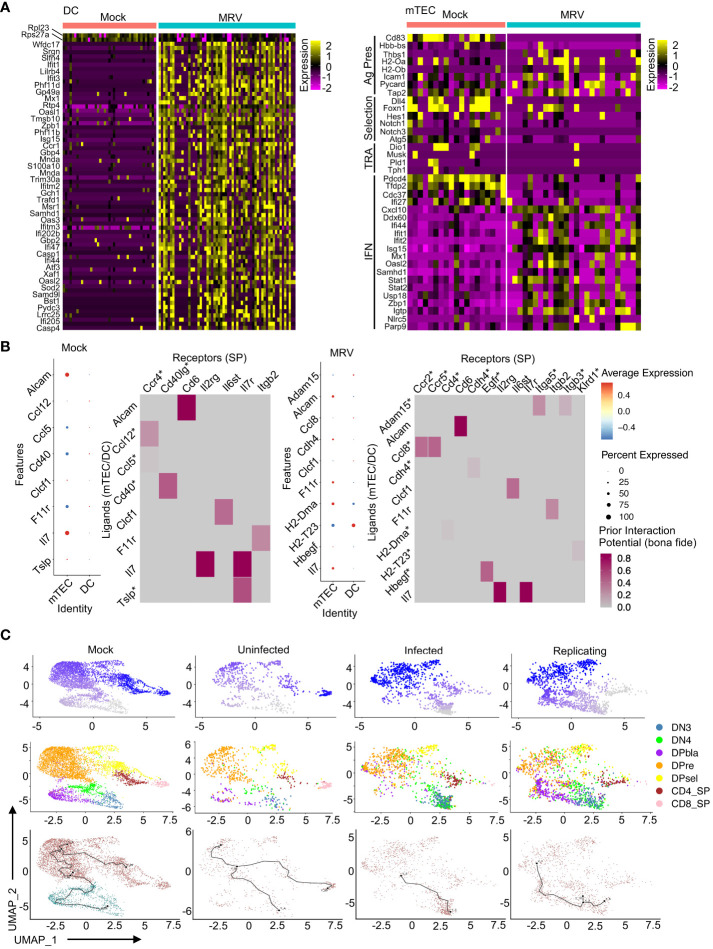
MRV infection results in transcriptional alterations in DC and mTECs that are associated with disruption of thymocyte development. **(A)** Heat maps of DEGs in DCs and mTECs for Mock vs. MRV. **(B)** Nichenet analysis of SP receivers and mTEC/DC senders. Dot plots show potential ligands split by cell type. Heat maps show the strength of potential ligand-receptor interactions. Ligands correspond to SP and receptors to mTECs/DCs. Asterisk represents interaction unique to mock or MRV. **(C)** Pseudotime analysis of developing thymocytes split by Mock or MRV uninfected, infected, or replicating. The first row is composed of pseudotime plots. The second row is UMAPs of cell types. The third row is the roots and nodes used to generate the pseudotime.

DEG analysis of DCs showed that MRV infection led to downregulation of *Rpl23* and *Rps27a*, ribosomal proteins that have been shown to be involved in dendritic cell activation, antigen presentation and cytokine production (**Figure 7A**) (88, 105). A greater number of genes are upregulated in DCs after MRV infection, many of which are involved in the innate immune response (i.e. *Ifit1, Ifit3, Mx1, Gbp2, Oasl1, Isg15, Ccr1, Ifitm2, Oaxl2, Casp1, Casp4*) that would be predicted in response to a viral infection (**Figure 7A**). In mTECs, we observed an overall downregulation of genes involved in antigen presentation, Notch signaling/selection, and TRAs after MRV infection. Genes involved in negative regulation of type I IFN (*Pdcd4, Tfdp2, Cdc37, Ifi27*) were downregulated while genes involved in or reflective of increased IFN signaling were upregulated (**Figure 7B**). These data suggest that antigen presenting cells that contribute to central tolerance demonstrate transcriptomic dysregulation during neonatal MRV infection.

Our previous work established a potential link between neonatal disruption of central tolerance by MRV infection and development of autoimmunity later in life (2). As MRV infection induced transcriptional disruption in both thymocytes and the antigen presenting cells that mediate thymocyte selection, we utilized network analysis to identify interactions between DP and SP thymocytes and antigen presenting cells within the thymic environment (106). We found that mTECs demonstrated the strongest signal of interaction with SP cells in the mock and MRV infected thymus (**Figure 7B**). Interestingly, in SP thymocyte to mTEC/DC analysis, MRV infection resulted in a loss of the *Ccl12/Ccl5:Ccr4* and *Cd40:Cd40lg* potential interaction and a gain of *Adam15:Itga5/Itgb3, Ccl8:Ccr2/Ccr5, Cdh4:Cdh4, H2-Dma: Cd4, H2-T23:Klrd1*, and *Hbegf: Egfr* potential interactions (**Figure 7B**). Similarly, MRV infection resulted in putative alteration in interactions between cTECs and DP cells that included a potential loss of the *Ptn: Ptprz1* interaction and a gain of *Cyr61:Itgam, Hbegf: Egfr, Lamb1:Itga6, Plau: Plaur*, and *Sema3f:Plxna3*, among other interactions (**Supplementary Figure 4**). Moreover, pseudotime analysis of thymocytes suggested that MRV infection and replication resulted in disruption of the transition of DN to DP cells and DP to SP cells that was not observed in the mock infection or in uninfected cells from the MRV infection sample (**Figure 7C**). Taken together, these data demonstrate infection and transcriptomic dysregulation of DCs and mTECS, and altered DC and mTEC interactions with DP and SP thymocytes that results in disruption of normal thymocyte development.

### Pseudokinetics reveals patterns of gene expression based on cell type and infection status

**Figure 8:**
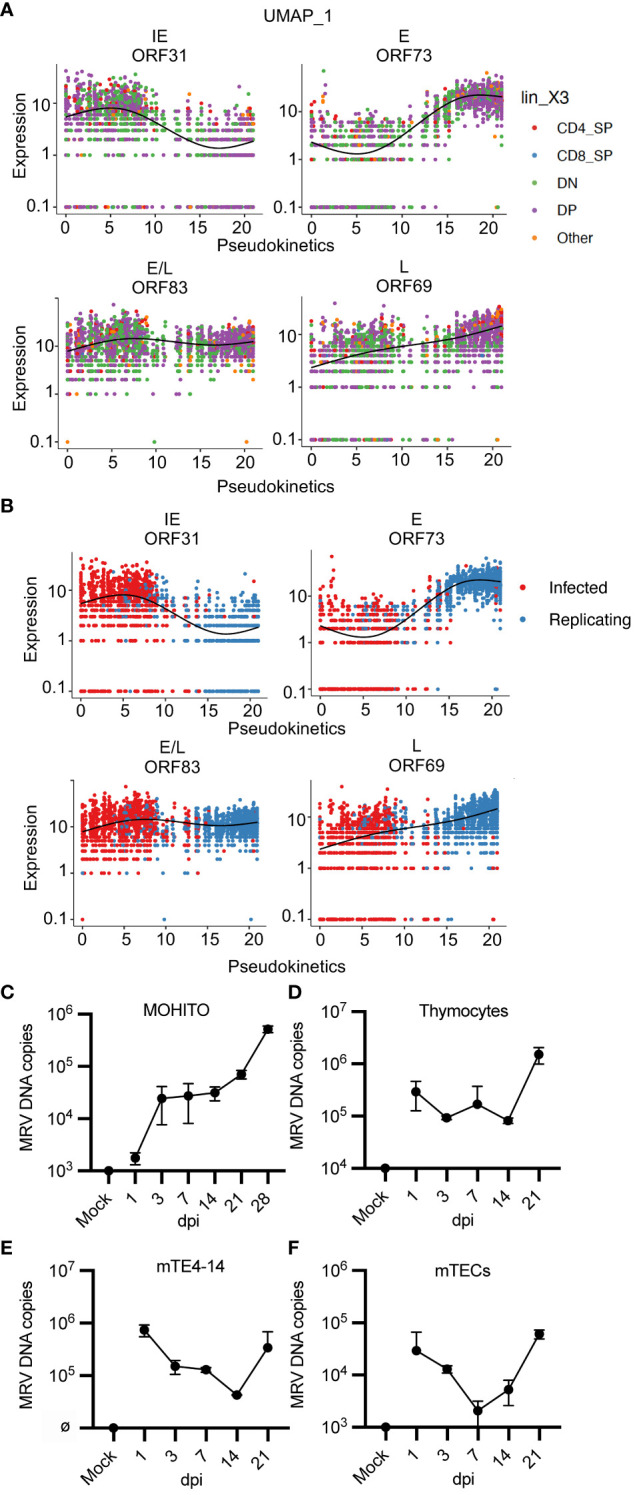
“Pseudokinetics” analysis demonstrates ORF kinetic patterns and MRV replicates *in vitro* in a T cell line, thymocytes, and mTECs. **(A, B)** Plots show expression of representative ORFs in each stage of infection, colored by **(A)** cell type and **(B)** infected vs. replicating status. **(C-F)** Cells were infected with MRV (1 genome/cell into 2.5e5 cells), performed in duplicate, and harvested at the stated time point as dpi compared to mock (uninfected at day 0) and input. Cells were washed after 24 hpi before collected of cells. MRV DNA was analyzed by qPCR and represented as MRV DNA (genome) copies per 2.5e5 cells for **(C)** MOHITO cells, **(D)** primary thymocytes, **(E)** mTE4–14 cells, and **(F)** primary mTECs. Error bars represent standard deviation.

MRV genes were assigned to a kinetic phase based on homology to HHV-6 and HCMV, although 55 genes had unknown kinetics due to unclear homology or homology to uncharacterized genes. We used pseudotime analysis of MRV ORFs to determine expression levels by thymocyte type or by infection status, which we termed “pseudokinetics.” The IE *ORF31* was expressed in most thymocytes, but expression was highest in cells classified as infected (**Figures 8A, B**). This pattern was observed in all IE genes (**Supplementary Figure 5A**). A representative E and a representative L gene, *ORF73* (major capsid protein) and *ORF69*, both demonstrated the highest expression in DP and CD4SP, as well as cells assigned as replicating compared to infected (**Figures 8A, B**). E/L gene, *ORF83* appeared biphasic and was present in all cell types and infection status, comparable to what was observed for its HCMV homologue, UL97 (**Figures 8A, B**) (82). While the majority of E, E/L and L genes showed increased expression over the course of progression from infected to replicating, some genes demonstrated stable or decreasing expression (**Supplementary Figures 5B**, **6**). Some of the MRV ORFs with unknown kinetics showed low or no expression. *ORF2, 13, 108, 112, and 115* showed a trend of decreased expression in cells designated as replicating vs infected. Many of the other ORFs demonstrated pseudokinetics consistent with E, E/L or L kinetics in which expression increased upon progression from infected to replicating (**Supplementary Figure 7**). By establishing pseudokinetics based on ORF expression level by cell type and infection status, we identified patterns of expression that suggest the kinetic stage of expression.

We next evaluated MRV ORF expression by either infection or replicating status. We grouped ORFs by putative kinetics based on homology or as genes with unknown kinetics. There were 12 MRV ORFs that demonstrated increased relative expression in cells designated as infected compared to replicating (**Supplementary Figures 1B**, **6**, **7**). This included genes with predicted kinetics based on homology to HHV-6 and/or HCMV (*ORF30, 31, 102, 110, 14–18* and *78*) as well as genes with unknown kinetics (*ORF2* and *ORF108*), suggesting that these ORFs have shared kinetics (**Supplementary Figures 1B**, **7**). Most of the remaining genes with kinetics predicted by homology were expressed at higher levels in cell designated as MRV replicating, while some of the unknown genes also demonstrated increased expression in cells designated as replicating (*ORF11–13, 35, 36, 38, 60, 68, 70, 75, 76, 81, 82, 101, 111, U25, U32*) (**Supplementary Figure 1B**, **7**). Finally, we performed hierarchical analysis of MRV ORFs which identified three distinct groups of cells with a differential pattern of ORF expression that were mostly aligned to infection status (**Supplementary Figure 1C**). Together, these approaches demonstrated features of MRV ORF expression that are useful in predicting the stage of infection of a given cell as well as expression kinetics of uncharacterized herpesvirus ORFs.

### *In vivo* tropism identifies *in vitro* cell culture systems that support MRV replication

A cell culture system that supports MRV replication has yet to be established despite testing in a broad range of cell culture systems [(62), negative data from our studies]. Our scRNAseq studies suggested that thymocytes at the DN3, DN4, DP and CD4SP stages of development, as well as mTECs, are susceptible to MRV infection and replication (**Figures 1C**, **5B**). We therefore cultured a DP T cell lymphoblastic leukemia cell line (MOHITO) (98), primary thymocytes, an immortalized mTEC cell line (mTE4–14) (99), and primary mTECs. Cells were infected with 1 genome per cell and viral DNA levels were measured over a time course to establish a growth curve. In all types tested, we observed an increase in viral DNA copies per cell starting between 7 and 21 dpi (**Figures 8C–F**). These results confirm patterns of tropism identified by our scRNAseq approach and establish primary thymocytes and mTECs, as well as related cell lines, as cell culture systems susceptible to MRV infection and replication.

## Discussion

In this study we have characterized the tropism of MRV in the thymus and identified the host transcriptome during acute, neonatal infection using scRNAseq. We utilized the pattern of MRV ORF expression, including robust expression of replication dependent L genes, to identify cells that appeared to be infected with MRV compared to cells that were supporting MRV replication (84). We found that thymocytes at the DN, DP, and SP stages were susceptible to MRV infection and replication, however DN1 and 2, and CD8SP appear only minimally susceptible to infection. This correlates with our previous work demonstrating that DP and CD4SP cells undergo a rapid decline in cell count between days 3 and 7 post infection (**Supplementary Figure 1A**) (2, 15, 16), although whether these cells are depleted due to direct infection, killing by antiviral CD8 T cells, or signaling induced apoptosis remains unknown. We found that ETPs and some non-thymocyte cells, including mTECs and DCs, appear to be susceptible to MRV infection and/or replication (**Figure 6**). We also identified patterns and kinetics of viral gene expression that are characteristic of the cell’s MRV infection status. By comparing cells at different stages of the viral replication cycle, we found broad transcriptional alterations in MRV-infected and uninfected bystander cells after neonatal MRV infection. The differential gene expression suggested that pathways typical of herpesvirus infection such as the inflammatory response, cell cycle control, and cholesterol biosynthesis were impacted by infection. Pathways necessary for thymocyte development were also affected, demonstrating a tissue-specific effect of MRV infection. Finally, our scRNAseq approach to identifying viral tropism facilitated the establishment of several cell culture systems that support MRV replication *in vitro*.

While there are several examples of single-cell transcriptomic approaches that have been utilized to identify viral tropism (107–109), identification of herpesvirus tropism during acute, *in vivo* infection of an entire organ has not yet been performed. Our study demonstrates that by using late gene expression and overall viral ORF expression patterns, tropism and infection status can be evaluated at a single-cell level. This approach to studying tropism is especially useful in the case of viruses in which there are limited molecular tools, as had been the case for MRV. The human roseoloviruses can infect a wide range of cell types *in vivo* and *in vitro*, including T cells (especially CD4+ T cells), T cell lines, thymocytes, B cells, macrophages/monocytes, epithelial cells, fibroblasts, and neuronal cells (30, 110–113). We similarly established that MRV infects thymocytes starting at the DN3 stage through the CD4SP stage, ETPs, and thymic epithelial cells. It is of interest that we did not observe MRV gene expression in cTECs, despite previous studies showing that cTEC numbers transiently decrease after neonatal infection, suggesting that cTECs survival or proliferation may be altered by the inflammatory response to MRV infection (2). Additionally, for dendritic cells, only two cells showed robust expression of most MRV genes. While this may represent phagocytosis of infected cells, the lack of viral genes identified in contemporaneous monocytes/macrophages argues against this possibility. Future studies that enrich for these minority cell types in the thymus could be pursued to examine this lower percentage susceptibility. Importantly, we validated the predicted tropism from our scRNAseq analysis with *in vitro* cell culture systems. We found that thymocytes, a T cell line, mTECs and an mTEC cell line were all susceptible to infection. These novel tools offer new opportunities to study viral kinetics, the host-virus relationship, and the molecular virology of MRV.

Our results suggest that there are shared and differential effects of MRV at each thymocyte stage. Thymocyte maturation is dependent on specific maturation signaling, TCR rearrangement and signaling, cytokine signaling, various cell surface molecules, metabolism, as well as modulation of the cell cycle and apoptosis machinery (33, 34). In DN, DP and CD4SP cells, MRV infection resulted in altered expression of genes involved in DNA replication, transcription, cell cycle, metabolism, type II IFN and TNF signaling, and DNA repair (**Figures 2D**, **3D**, **4B**, **5**). While these pathways are commonly altered by herpesvirus infections and may be a result of direct infection, a portion of cells are uninfected and MRV-mediated changes in the microenvironment could also impact uninfected cells. For example, NF-κB, TLR, and type I IFN signaling have been shown to impact T cell maturation in the thymus (114–119). Regarding TNFα and NF-κB pathway stimulation, prior work has shown that HHV-6 infection of CD4+ T cells results in apoptosis of infected, and likely neighboring, uninfected cells, through a TNFα and NF-κB dependent process (120, 121). Evaluation of the cytokine signature based on the Immune Response Enrichment Analysis demonstrated a strong type I IFN response in all thymocyte stages (104). In DN and CD4SP, IL-7 response was also represented. It was of interest that all cells, but notably DN, DP, and CD4SP, featured a signature suggestive of IL-36a signaling. IL-36 is a member of the IL-1 superfamily that, along with IL-1 and IL-18, have been shown to mediate T cell activity (122, 123). While IL-36 has been shown to potentiate type I IFN signaling in response to herpes simplex virus-1, its role in beta-herpesvirus response or in the thymus remains poorly understood and could represent an important target in MRV-induced thymus dysfunction (124).

Other pathways necessary for thymocyte maturation were notably altered by MRV infection. For example, during the maturation of DN to DP, thymocytes undergo 6–8 divisions, something that would likely be impacted by the alterations in cell cycle (i.e. G1 to S cell cycle control) observed in MRV infected cells (125). Additionally, we observed upregulation of genes involved in cholesterol biosynthesis, a process known to be important for beta-herpesvirus replication and T cell function but less well studied for thymocyte development (74, 126). We also noted downregulation of genes known to be essential for maturation of DN and DP cells, including *Cd4, Cd8, Rag1, Rag2, Tcf7, Themis, Lat, Rorc, Lck, Zap70, Cd5, Cd27, CD28, Bcl11b, Tox*, and genes in the PluriNetwork pathway that are involved in pluripotency and cell fate (42, 50, 54, 102, 127–132). The thymocyte-expressed molecule involved in selection (Themis) has been well characterized as a major player in the maturation and selection of thymocytes (128). Themis interacts with Lck, Zap-70, and Lat to alter TCR and Lat signaling during positive and negative selection, both of which are impaired in *Themis-/-* mice (128, 133–135). These findings suggest that in addition to direct infection, disruption of pathways necessary for thymocyte survival and differentiation could contribute to thymocyte depletion and altered selection during MRV infection. Ongoing studies targeting the genes and pathways perturbed by MRV infection will provide additional data defining the specific mechanism of MRV mediated DP and CD4SP depletion as well as loss of central tolerance.

Thymocyte selection is mediated by antigen presenting cells such as thymic dendritic cells, cTECs and mTECs (58). Our prior studies demonstrated that neonatal MRV infection results in a transient a reduction in the number of each cell type and the reduced expression of the transcriptional regulator, *Aire*, as well as TRAs, suggesting a possible mechanism of negative selection disruption and escape of autoreactive T cells (2). It was thus interesting to find that MRV appears to directly infect mTECs (**Figure 6**). The impact of infection appears to be both direct and indirect since we observed transcriptional perturbations in infected and uninfected cells compared to mock infection (**Figure 7B**). For example, *Dll4, Hes1, Foxn1, Notch1, and Notch2*, which all have important roles in mTEC and/or thymocyte maturation, were downregulated by MRV infection (35, 45). We did not identify transcripts of TRAs specific to stomach antigens or TRA expression regulators (*Aire, Fezf2*). This is likely due to the overall low transcript level of these genes and because mTECs represent a minority population in our sample. We did observe reduced expression of several TRAs, and reduced expression of genes involved in antigen presentation, both of which are necessary for negative selection (60). Finally, there was an overall reduction in expression of genes that antagonize the type I IFN response and an increase in type I IFN responsive genes (**Figure 7B**). IFN signaling is suggested to impact mTEC function and survival (115, 116, 118, 119, 136). All these findings provide clues regarding how MRV disrupts central tolerance.

Using NicheNet evaluation, we identified expression of receptor-ligand pairs that suggest cell-cell interactions within the thymus microenvironment. It was interesting to note that that there were interactions that were unique to the mock and MRV samples. In fact, the differential interactions give potential evidence as to how MRV impacts mTEC and DC interactions with SP cells, and cTEC interactions with DP cells. Two interesting potential interactions were present in mock but not MRV infection: *Ccl12/Ccl5:Ccr4* and *CD40:CD40lg.* CD40 and CD40L contribute to mTEC development and thymocyte development and tolerance (137, 138). In MRV infection, there was a potential gain in Ccl8:Ccr2/Ccr5 interaction. Chemokine signaling impacts thymocyte development and migration within the thymus and could offer insight into a mechanism by which MRV alters thymic function (139). Increased interaction between MHC H2-Dma and H2-T23 with CD4 and Klrd1 on CD4SP and CD8SP, respectively, could alter cell activation or selection. Regarding the potential EGF: EGFR interaction, EGF signaling plays a role in stromal cell regulation and T-cell differentiation, modulates fetal thymic growth and morphogenesis, and impacts the ability of TECs to sustain thymocyte differentiation *in vitro* (140). For the cTEC: DP evaluation, the *Ptn (pleiotrophin)-Ptprz1* interaction is observed in mock but not MRV infection. *Ptn* is expressed during TEC development and it impacts hematopoietic cell proliferation, development, and adhesion (96, 141). Based on expression data, there were several potential interactions that were gained in the MRV samples. *Sema3f*, for example, when expressed on thymocytes and TECs inhibits thymocyte migration by blocking CXCL12 and spingosine-1-phosphate -induced migration (142). Altered *Plau* expression results in decreased thymic Treg development (143). *Itga6* expression in TECs regulates expression of cell migration and immunologic synapse related genes (144). *Cyr61* expression on TECs boosts progenitor homing (145), but how the differential interaction with *Itgam* would impact thymocyte development is less clear. The findings in our studies provide known and novel receptor-ligand targets to understand how MRV influences antigen presenting cell-thymocyte interactions.

Recent single-cell transcriptomic studies of HCMV infection of cell culture suggested that the IE, E, and L classification based on a simple cascade of viral gene expression may not be wholly representative of the complex nature of herpesvirus expression kinetics and that some genes may be regulated by independent modules (82). Although our studies were performed at a single time point, using pseudotime analysis we identified differences in expression levels based on infection status (infected vs replicating), which we have termed pseudokinetics. Our results show that expression of genes assigned to IE, E, E/L, and L, based on homology, showed similar pseudokinetic expression patterns as those described in transcriptomic studies of HCMV (80, 82, 86, 146). Analogous to findings in HCMV, some MRV ORFs display expression pseudokinetics that suggests potential independent modules that contribute to their expression pattern. In our analysis, there appears to be a clear transition of MRV gene expression during progression from MRV infection to MRV replication, which is characterized by downregulation of the IE genes *ORF30* and *31*, and upregulation of the majority of E, E/L and L genes.

One unexpected pattern of expression was the downregulation of several ORFs upon transition from infected to replicating. Most notable of these were *ORFs 14–18*, which appear to be US22 family whose homologues (*U2* and *U3* for HHV-6, *UL23–25* for HCMV) have diverse roles in tegument-mediated transactivation, control of type I IFN response, and modulating cell cycle (147–150). This could suggest that these genes contribute to initiation of lytic infection in different cell types. Utilizing our studies, we can predict the kinetics of MRV genes that have no homology or are homologous to uncharacterized genes. Although we do not have a similar study in human roseoloviruses to compare our results, datasets from RNAseq to evaluate HHV6B gene expression in blood, tumors, and cell lines, as well as single-cell analysis CAR T cells and in a case report of DRESS could be analyzed in future studies to assess expression patterns using the techniques we employed in this study (81, 83, 151). Overall, our work provides a foundation to understand herpesvirus gene expression *in vivo* during acute infection at the transcriptional level, and provides insight into the dynamics of viral gene expression over the course of infection.

The results of this study establish an atlas of roseolovirus tropism, gene expression, host-virus interactions, and host transcriptome disruption during acute, neonatal infection. The data generated from this study provide a framework to understand how thymic infection by MRV, and perhaps the homologous human roseoloviruses, results in loss of central T cell tolerance and subsequent autoimmune disease. Applying our data to studies of roseolovirus infections in patients, such as those undergoing thymectomy early in life due to congenital heart disease or patients undergoing thymus transplant for thymic deficiency, could identify the role of roseoloviruses, and potentially other viruses, in altering thymus function. Taken together, our studies demonstrate unique patterns of roseolovirus gene expression, host-virus interactions, and disruption of pathways necessary for thymocyte survival and selection in the thymus during acute, neonatal infection that could be explored to identify the mechanism and therapeutic targets of roseolovirus-induced autoimmunity.

## Data availability statement

The data presented in the study are deposited in the NCBI Gene Expression Omnibus repository, accession number GSE255738.

## Ethics statement

The animal study was approved by The IACUC of Washington University in St. Louis. The study was conducted in accordance with the local legislation and institutional requirements.

## Author contributions

AB: Writing – review & editing, Writing – original draft, Visualization, Validation, Software, Methodology, Investigation, Formal analysis, Data curation, Conceptualization. EX: Writing – review & editing, Methodology, Investigation. BC: Writing – review & editing, Methodology, Investigation. ER: Writing – review & editing, Software, Methodology, Investigation, Formal analysis. MP: Writing – review & editing, Writing – original draft, Visualization, Validation, Supervision, Software, Resources, Project administration, Methodology, Investigation, Funding acquisition, Formal analysis, Data curation, Conceptualization. TB: Writing – review & editing, Writing – original draft, Visualization, Validation, Supervision, Software, Resources, Project administration, Methodology, Investigation, Funding acquisition, Formal analysis, Data curation, Conceptualization.

## Supplementary material

Supplementary Figure 1 MRV-induced CD4+ thymocyte depletion and MRV ORF expression kinetics. **(A)** Mice were mock or MRV infected on DOL 0 and the thymus was harvested at the designated time point. Flow cytometry plots showing CD4 and CD8 at each time point with graphs of absolute number of CD4SP thymocytes at each time point. **(B)** Heat map of ORFs comparing expression between Infected and Replicating, ordered by putative kinetics. **(C)** Hierarchical heat map of MRV recreates uninfected/replicating/infected populations.

Supplementary Figure 2 Transcriptional dysregulation of DN and DP populations after neonatal MRV infection. **(A-F)** Pathway enrichment for DN and DP subpopulations comparing Mock and MRV.

Supplementary Figure 3 Thymocytes demonstrate upregulation of genes suggestive of type I IFN and IL-36 signaling. **(A-D)** Spiral plots of cytokine expression based on MRV DEGs for each thymocyte type. Figures were generated with the Immune Dictionary App. Coloration was based on p-values, and bar length represents the enrichment score.

Supplementary Figure 4 MRV induces transcriptomic alterations that suggest altered interactions between DP thymocytes and cTECs. Nichenet analysis of DP receivers and cTEC senders. Dot plots show expression of potential ligands. Heat maps show the strength of potential ligand-receptor interactions. Ligands correspond to DP and receptors to cTECs. Asterisk represents interaction unique to mock or MRV.

Supplementary Figure 5 “Pseudokinetics” plots for **(A)** Immediate Early and **(B)** Early, ORFs colored by cell type.

Supplementary Figure 6 “Pseudokinetics” plots for **(A)** Early/Late and **(B)** Late, ORFs colored by cell type.

Supplementary Figure 7 Pseudokinetic and expression analysis of genes with unknown or uncharacterized homology **(A)** “Pseudokinetics” plots for Unknown, ORFs colored by cell type. **(B)** Infected vs. replicating heat map of ORFs with unknown kinetics.
